# Successful Treatment of Well-Developed Accessory Lower Limb Associated with Spinal Dysraphism

**DOI:** 10.29252/wjps.9.1.73

**Published:** 2020-01

**Authors:** Muhammad Saaiq, Faridullah Khan Zimri, Khaleeq-uz Zaman

**Affiliations:** 1 Department of Plastic Surgery, National Institute of Rehabilitation Medicine (NIRM), Islamabad, Pakistan;; 2 Department of Orthopedic Surgery, National Institute of Rehabilitation Medicine (NIRM), Islamabad, Pakistan;; 3 Department of Neurosurgery, National Institute of Rehabilitation Medicine (NIRM), Islamabad, Pakistan

**Keywords:** Accessory lower limb, Spinal dysraphism, Lipomyelomeningocele, Spina bifida

## Abstract

Accessory lower limb with spinal dysraphism are amongst the rarest known anomalies. We successfully managed a 5-months old female infant with surgical ablation of the accessory lower limb and repair of the associated large lipomyelomeningocele. A comprehensive review of the relevant literature was undertaken and presented herein. A classification system for accessory lower limb is also proposed.

## INTRODUCTION

Accessory lower limb associated with spinal dysraphism constitutes an extremely rare congenital anomaly. The exact underlying mechanisms involved in the embryogenesis still continue to be explored. Several hypotheses have been presented to provide possible explanation in this regard.^1^^-^^4^ For instance, it may be the result of a primary mesodermal defect involving the paraxial mesoderm. A famous hypothesis of conjoined twinning has also been proposed in the past. Failure of regression of limb bud of a conjoined twin is thought to account for the accessory lower limb. Since there is an underlying process of deranged embryogenesis, the anomalous condition may be associated with other congenital anomalies such as spina bifida, vertebral body malformations, anorectal malformations and genital anomalies.^1^^-^^4^

A plethora of confusing terms has been employed to denote the anomaly of accessory lower limb in the previously reported cases. There is dire need to turn this chaos into order and develop a uniform and comprehensive terminological approach, as well as meaningful classification system. This will not only help the future surgeons to better plan their surgical strategy, but also help to reduce their stress of unknown. Additionally, this will help the future researchers to carry out more yielding research pursuits on the subject.^1^^-^^4^ To the best of our knowledge, only a handful of such cases have been reported in the published literature.^5^^-^^18^ This rarity prompts us to share our experience.

## CASE REPORT

A 5-month old female infant was brought by worried parents to our department with an accessory lower limb attached to the lower back. The infant was born with it and it was growing commensurate with the growth of the rest of the infant’s body. The infant was the fourth child of her parents, who had contracted a non-consanguineous marriage ten years ago. All the previous siblings were normal. The parents did not give any history of maternal illness, smoking, alcohol abuse, medication intake, radiation exposure or any other obvious gestational insult through the course of their pregnancy that yielded the anomalous baby. 

**Fig. 1:**
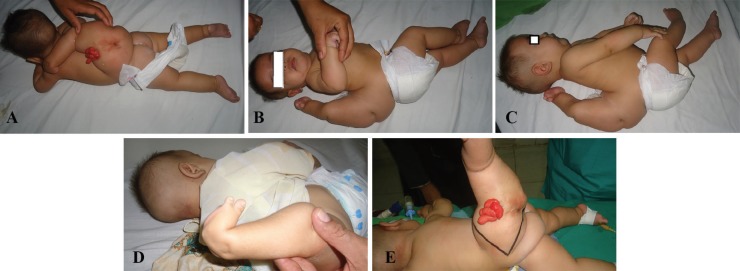
**A-E: **The accessory lower limb was arising from the lower back at the level of L2 to S1. It was pointing cranially. Grossly, it resembled a normal lower limb with a partially developed foot containing only big toe and the next adjacent toe. The accessory limb was bearing four blind ending gut loops of red color. These used to wet the clothing with their watery secretion. Additionally, there were areas with pitting and dimples, mimicking the natal cleft and anus

The mother had not undergone any antenatal screening and hence, the condition was obvious only after natural delivery at home. Except for sluggish movement in the normal right lower limb, the infant had no other associated systemic abnormality. The parents were particularly concerned about the gruesome appearance and difficulty with care and posture of the infant. Clinical examination of the infant revealed a well-developed accessory lower limb arising from the back at the level of L2 through S1 vertebrae (Figure 1A-D). 

The accessory lower limb was pointing cranially. Grossly, it resembled a normal lower limb with a partially developed foot containing only big toe and the adjacent next toe. On the accessory lower limb, there were four blind ending gut loops of red color (Figure 1E). These used to wet the infant’s clothing with their watery secretion. Additionally, there were areas with dimples mimicking the natal cleft and anal opening. There was no spontaneous movement, however withdrawal to pain was present in the accessory lower limb. 

**Fig. 2:**
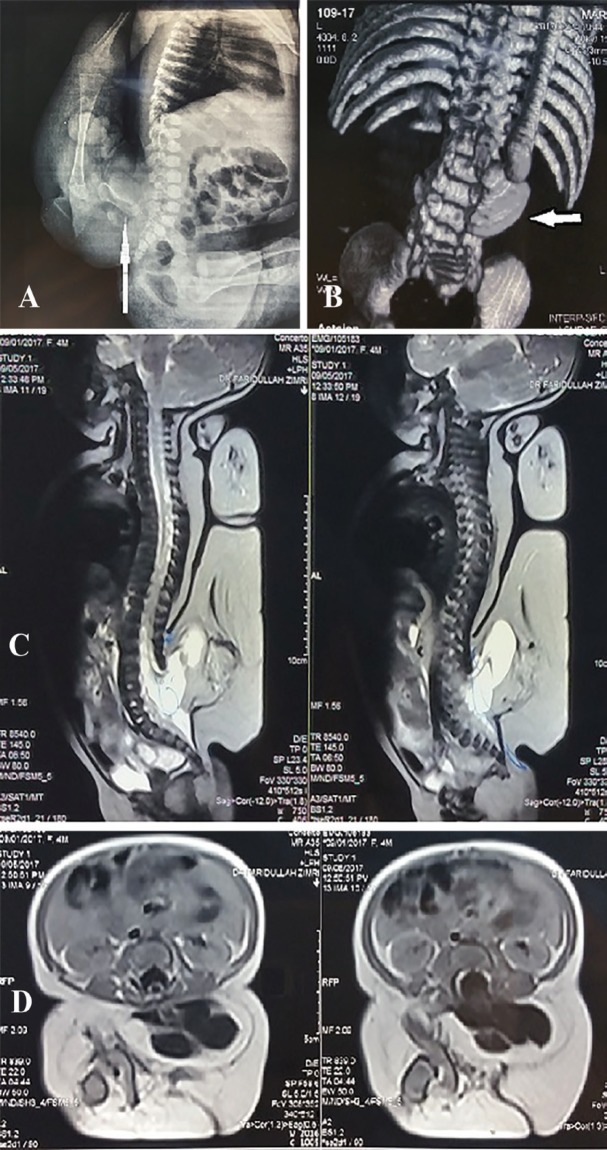
**A:** Plain X-rays showed the osseous components of the accessory lower limb. The arrow is pointing towards the rudimentary hemi-pelvis. **B:** Lumbosacral computed tomography with three dimensional reconstruction revealed deficient posterior bony elements at the level of L2 through SI vertebrae along with evidence of lipomyelomeningocele. It also confirmed an accessory lower limb attached to the right gluteal muscles. The limb had a rudimentary iliac bone (the arrow is pointing towards it), femur, tibia, fibula, rudimentary foot bones, hip and knee joints. **C:** MRI lumbosacral spine, sagittal view. **D:** MRI lumbosacral spine, axial view. The whole spine Magnetic resonance imaging (MRI) confirmed the presence of spinal dysraphism involving lumbar spine with deficient posterior elements. The spinal cord and thecal sac were protruding posteriorly and towards the left superiorly through the defect in the posterior elements at the level opposite L3 through S1 vertebrae, revealing a large lipomyelomeningocele measuring approximately 4.7×3×4.8 cm. The CT scan findings of the accessory lower limb were re-confirmed

The normal left lower limb was neurologically intact. However, there was grade 3 weakness of the normal right lower limb. The infant had normal looking external genitalia, anus, abdominal and thoracic walls. Plain X-rays showed the osseous components of the accessory lower limb (Figure 2A). Lumbosacral computed tomography with three dimensional reconstruction revealed deficient posterior bony elements at the level of L2 through SI vertebrae along with evidence of lipomyelomeningocele. 

It also confirmed an accessory lower limb attached to the lumbosacral spine and right gluteal muscles. The limb had a rudimentary iliac bone, femur, tibia, fibula, rudimentary foot bones, hip and knee joints (Figure 2B). Whole spine magnetic resonance imaging (MRI) confirmed the presence of spinal dysraphism involving lumbar spine with deficient posterior elements (Figure 2C and D). The spinal cord and thecal sac were protruding posteriorly and towards the left superiorly through the defect in the posterior elements at the level opposite L3 through S1 vertebrae, revealing a large lipomyelomeningocele which measured approximately 4.7×3×4.8 cm. 

**Fig. 3:**
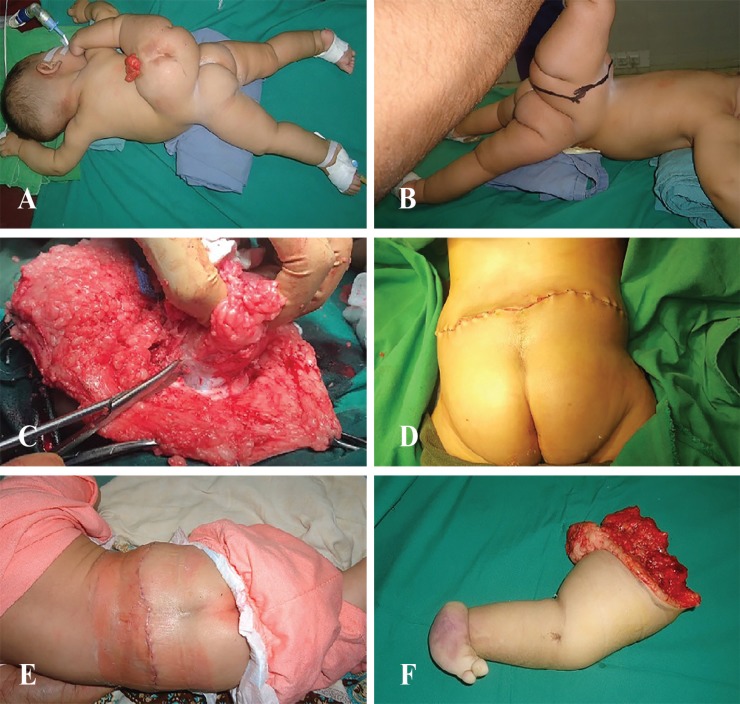
**A: **The infant was positioned prone on the operating room table for undertaking surgery. **B:** An elliptical skin incision with transverse orientation was designed to ensure adequate closure of the resultant defect after limb extirpation. **C:** The neural placord was carefully dissected, de-tethered from attachments and returned. Dural closure was performed. The overlying dorsolumbar fascia was closed. **D:** The skin was closed with subcuticular absorbable sutures. Steristrips were applied to support the wound. **E: **The wound was perfectly fine on the first dressing change on 5^th^ postoperative day, after removal of steristrips. **F:** The ablated accessory limb

The CT scan findings of the accessory lower limb were also re-confirmed. The cranial magnetic resonance was normal with no associated cerebral anomalies found. Written informed consent was taken from the parents of the infant for undertaking surgery and taking serial photographs through the course of treatment. At the time of surgery, the patient was positioned prone on table (Figure 3A). An elliptical skin incision (transversely oriented on the lower back) was designed to ensure adequate closure of the resultant defect after limb extirpation (Figure 3B).

The limb was eschmarked to preserve blood and circulatory physiology of the infant during surgery. Following incision of the skin and subcutaneous tissues, deeper dissection was undertaken in a standard fashion and carried down to the fibro-osseous attachments of the rudimentary hemi-pelvis. The accessory lower limb was ablated into by disarticulation at the level of the accessory hemi-pelvis/ileum, which was carefully dissected from its attachments to the lumbosacral spine and gluteal muscles. Dissection and repair of the large lipomyelomeningocele was performed in a standard fashion. The neural placord was carefully de-tethered from attachments and returned. 

The overlying dorsolumbar fascia was closed in double breasting manner. The skin flaps were approximated in a tension free manner (Figure 3C). The wound was closed with subcutaneous absorbable sutures and steristrips were employed (Figure 3D). The immediate postoperative period was uneventful and the infant was discharged home on 5^th^ postoperative day (Figure 3E). Figure 3F shows the ablated accessory limb. She was clinically fine at her three months follow up visit with no additional neurologic deficits or any other complications. Her parents were instructed to ensure regular follow up visits at six months intervals. 

## DISCUSSION

Our patient was a female child. As far the literature search, the anomaly of accessory lower limb shows a predominant female predilection (Table 1). Overall the condition was four-folds more common among females than males. The possible explanation for the more frequent affliction of females by the anomalous condition is hitherto missing.^19^^-^^21^ In our patient, the accessory lower limb was attached to the lumbosacral region. Various attachment sites of the accessory lower limb have been reported by previous researchers.^19^^-^^21^

**Table 1:** Comprehensive review of publications on accessory lower limb associated with spinal dysraphism

**Publication**	**Number/ Gender**	**Condition of the accessory lower limb**	**Site of attachment of the accessory lower limb**	**Condition of the Spinal dysraphism**	**Other associated anomalies/ status**
Krishna *et al.*,^5 ^1989, India	1 F	Well-developed, with all the bony components including half of the iliac and pubic bones, femur, both leg bones, and bones of the foot.	Lumbosacral	Lipomyelomeningocele	Rudimental phallus and scrotum-like skin at the base of the accessory limb.
Nanni *et al.*,^6 ^1994, Italy	1 F	Moderately-developed, containing single long bone and four metatarsals with their respective phalanges.	Sacral/ Buttock	Myelomeningocele	Anorectal agenesis and rectovestibular fistula. Operated on the first day of life.
Krishna *et al.*,^7 ^1999, India	1 F 1 F	Mildly-differentiated lower limb like outgrowths on the lipomatous mass, containing single long bone.	Lumbar Lumbar	Lipomyelomeningocele Lipomyelomeningocele	Rudimentary external genitalia, bowel loops on the surface of the limb.
Gamanagatti *et al.*,^8 ^2003, India	1 F	Moderately-developed, only thigh with no parts distally. There was only iliac bone and femur.	Sacral	Lipomyelocele	Nil
Zhao *et al.*,^9^ 2006, China	1 M	Well-developed, articulating with the pseudo-pelvis through a hip and containing femur, tibia, fibula, and duplicated foot.	Lumbosacral	Lipomyelomeningocele	Weakness left lower limb, pseudo-phallus and navel.
Lende *et al.*,^10 ^2007, Ethiopia	1 F	Well-developed, with all the bony components including half of the iliac and pubic bones, femur, both leg bones, and the foot.	Lumbar	Myelomeningocele	Isolated colon loop.
Wasnik *et al.*,^11 ^2007, India	1 M 1 M	Poorly-developed accessory lower limb represented by bony struts attached to the ilium.	Lumbar L4-L5	Lipomyelomeningocele Lipomyelomeningocele	Delayed presentation at the age of 9 and 11 years. Weakness of normal right lower limb. Urinary incontinence, Non healing ulcers of foot
Khan *et al.*,^12 ^2010, Pakistan	1 F	Moderately-developed, predominantly showing leg and foot.	Lumbosacral	Meningocele	Successfully operated at the age of 4 months.
Murphy *et al.*,^13 ^2013, USA	1 M	Moderately developed, consisting of a short femoral segment, ﬁbular segment, single tarsal bone and three toes.	Lumbosacral	Lipomyelomeningocele	Delivered through un-complicated caesarean section. Underwent successful surgery at the age of 7 months.
Bayri *et al.*,^1 ^2014, Turkey	1 M	Well-developed , including rudimentary hemi-pelvis, femur, tibia, and a rudimentary foot containing only one toe.	Lumbosacral, L5S1	Meningocele	Delivered through caesarean section.
Wilkes *et al.*,^14 ^2015, Honolulu USA	1 F	Poorly-developed, osseous limb, articulating with sacrum extending through the subcutaneous fat.	Lumbosacral L4S2	Lipomyelomeningocele	Nil
Nadeem *et al.*,^15 ^2016, Pakistan	4 F 1 M	Well-developed accessory lower limbs.	Lumbosacral	Lipomeningocele in one case, spina bifida among all.	Srotum and phallus like structures, gut like mucosa, a right club foot, one each.
Awad *et al.*,^16 ^2016, Egypt	1 F	Well-developed accessory lower limb including well-formed foot, tibia, fibula, knee joint, femur, atrophic ischial bone and a buttock like appearance.	Bifid spine at D12-L3 level	Lipomatous mass with spina bifida	Rudimentary intestinal loop.
Bodeliwala *et al.*,^17 ^2017, India	1 F	Mildly-developed, predominantly showing a partially formed foot with syndactylized toes and nails.	Lumbar L2-3	Meningomyeloocele	Left club foot.
Priyawansha *et al.*,^18^ 2018, Sri Lanka	1 M	Moderately-developed , consisting of femur and a rudimentary foot.	Thoracolumbar	Meningomyeloocele	Delivered through caesarean section because of obstructed labour. Umbilical hernia. Bilateral talipes equinovarus. Pre-auricular tags. Underwent surgery at the age of 22 days. Postoperatively he had CSF leak and lower limb weakness. Died on 8^th^ post operative day.
Current case Saaiq *et al.*, 2020, Pakistan	1 F	Well-developed accessory lower limb.	Lumbosacral L2-S1	Lipomyelomeningocele	Rudimentary loops lined by gut mucosa, areas with dimplings mimicking the natal cleft and anus. Grade 3 weakness of the normal right lower limb.

Early splitting of one limb bud may result in an accessory limb attached to lumbosacral region with associated spinal dysraphism. However, if the limb bud splits a little latter in the embryonic life, the accessory lower limb would be attached more distally. For instance, to the acetabulum, buttock or thigh rather than back. Among these cases, there will be no associated spinal dysraphism.^19^^-^^21^ Our patient had the closed variety of spinal dysraphism. These anomalies stem from malformations of the dorsal embryo that could be open or closed.^1^^,^^4^^,^^22^

The open dysraphisms are always associated with a Chiari II malformation. The spectrum of open dysraphisms include myelomeningocele, hemi-myelomeningocele, myeloschisis and hemi-myelocele. The closed spinal dysraphisms are characterized by presence of intact overlying skin and can present with or without a mass. The closed dysraphisms with mass include the spectrum of lipomyelomeningocele, meningocele, myelocystocele and lipomyeloschisis. The closed spinal dysraphism without a mass include complex dysraphic states, ranging from complete dorsal enteric fistula to neurenteric cysts, split cord malformations, dermal sinuses, caudal regression and spinal segmental dysgenesis, bony spina bifida, tight filum terminale, filar and intradural lipomas and persistent terminal ventricle.^1^^,^^4^^,^^22^

In our patient, the accessory lower limb was associated with lipomyelomeningocele. The published literature reports that the accessory lower limb is associated with spinal lipomas (i.e. lipomyelomeningocele, lipoma or lipomyeloce) in 50% of cases, with meningocele in 36% of cases and with myelomeningocele in 14% of cases. The lipomyelomeningocele represents a closed neural tube defect formed mainly due to a defect in primary neurulation in which mesenchymal tissue enters into neural placode and forms lipomatous tissue.^1^^,^^4^^,^^22^

The spinal cord becomes tethered to the fat and may then be pulled downward in the course of development as well as extruded from the vertebral canal into adjacent tissues. Various clinical impairments may develop. Based on a hypothesis, lipomyelomeningoceles are secondary neural tube defects that occur due to rupture of the neural tube under intact ectoderm. The leakage of the proteinaceous neural tube fluid acts as an abundant source of Schwann cells, which collect under the skin and dedifferentiate.^1^^,^^4^^,^^22^

The majority of the dedifferentiated cells develop into a lipomatous mass that may extend intradurally. Sometimes cartilage, bone, muscles and nerves may also develop in this lypomatus mass. On rare occasions, these components may grow in an organized manner and develop into an accessory limb. The stimulus for this organized growth remains uncertain.^1^^,^^4^^,^^22^^-^^24^ In our patient, we found ectopic gut loops on the accessory lower limb. Published literature has reported on association of accessory lower limb with anomalies of almost other systems. For instance, anorectal malformations, ectopic intestinal loops and accessory external genitalia (Table 1).^22^^-^^24^

MRI is the modality of choice, as it can assess other associated conditions like meningocele, myelomeningocele, lipomeningocele, split cord malformations, dermal sinuses and associated anomalies. CT scan with 3D reconstruction helps to get precise idea of the osseous components and thus helps with surgical planning.^22^^-^^24^ Hence, we recommend multimodal imaging studies for pre-operative evaluation and subsequent surgical planning. Our patient belongs to a far flung area of a remote province of the country (Balochistan) that lacked basic health care facilities. 

The patient’s mother had not undergone any antenatal evaluation and the delivery also occurred at home. These congenital accessory limbs can be easily diagnosed prenatally because of their apparent presence. We undertook surgery to ablate the accessory lower limb and perform repair of the large lipomyelomeningocele. It was our first time experience to undertake surgery for a complex case of this nature. The senior author (i.e. the neurosurgeon) organized and motivated the surgical team, anesthesia colleagues and the supporting staff. 

At the very outset, we had apprehensions regarding our surgical approach to the infant and the possible difficulties that we may encounter through the course of surgery. We were also fearful of possible complications such as the worsening of neurological condition postoperatively. We spent a good deal of time to plan our surgical strategy and carry out web-based search to review the relevant literature. To our pleasant surprise, we enjoyed a smooth and fast execution of the planned surgery. It took us only ninety minutes to complete the entire operation. Also, there was no additional neurological deficit to our great relief. 

Our patient had an uneventful post-operative course and we did not encounter any complications. In series of five cases, various postoperative complications such as wound dehiscence, CSF leakage and postoperative adhesions formation between spinal cord and dura along the scar line were shown.^15^ We undertook the surgery at the age of 5-month, because our patient presented at that age. The published literature does not indicate any consensus regarding the timing of surgery. The previously reported cases showed a wide variation in the timing of surgery, ranging from the age of one day to as late as early teens. 

During our literature search and review, we found that no uniform terminological approach exists towards the accessory lower limb. The condition has been given various names through the course of time. For instance, tripedus, polymelia, diplomyelia, heteropegus, rachypagus, rachypagus twin, parasitic rachypagus dysraphic appendages, aborted twinning, heterotopic redundancy, rudimentary or aborted accessory limb, caudal duplication and leg duplication. Based on the reviewed literature, the authors suggest a simple classification system based on the gross morphologic appearance and degree of differentiation of the accessory lower limb.^22^^-^^26^

It requires only clinical evaluation and imaging and is applicable across the entire spectrum of anomalous accessory lower limb (Table 2). In our study, accessory lower limb with spinal dysraphism was shown to carry significant distress for the parents. Adequately planned surgical ablation of the limb and repair of the associated lipomyelomeningocele were demonstrated to help to convent the grotesque deformity into a humanoid appearance.

**Table 2:** Morphologic classification for accessory lower limb

**1) Accessory lower limb with spinal dysraphism:**
a. Well-developed accessory lower limb.^1^,^5^,^9^,^10^,^15^,^16^
Grossly resembles the normal lower limbs and possesses all osseous components of the lower limb and pelvic girdle.
b. Moderately-developed accessory lower limb.^6^,^8^,^12^,^13^,^18^
Moderately sized as compared to the normal lower limbs. Some bony parts missing.
c. Mildly-developed accessory lower limb.^7^,^17^
Grossly smaller sized. Rudimentary isolated parts such as foot or a single long bone.
d. Poorly-developed accessory lower limb.^11^,^14^
Not showing resemblance to lower limb but has bony struts representing osseous elements of lower limb.
**2) Accessory lower limb without spinal dysraphism:** 19 **-** 21
a. Well-developed accessory lower limb.
b. Moderately-developed. accessory lower limb.^19^
d. Poorly-developed accessory lower limb.^21^
